# Human Virome in Cervix Controlled by the Domination of Human Papillomavirus

**DOI:** 10.3390/v14092066

**Published:** 2022-09-17

**Authors:** Thanayod Sasivimolrattana, Wasun Chantratita, Insee Sensorn, Arkom Chaiwongkot, Shina Oranratanaphan, Parvapan Bhattarakosol

**Affiliations:** 1 Medical Microbiology Interdisciplinary Program, Graduate School, Chulalongkorn University, Bangkok 10330, Thailand; 2 Center of Excellence in Applied Medical Virology, Department of Microbiology, Faculty of Medicine, Chulalongkorn University, Bangkok 10330, Thailand; 3 Center for Medical Genomics, Faculty of Medicine, Ramathibodi Hospital, Mahidol University, Bangkok 10400, Thailand; 4 Division of Virology, Department of Microbiology, Faculty of Medicine, Chulalongkorn University, Bangkok 10330, Thailand; 5 Department of Obstetrics and Gynecology, Faculty of Medicine, Chulalongkorn University, Bangkok 10330, Thailand

**Keywords:** virome, human papillomavirus, HPVs, cervical cancer

## Abstract

Although other co-viral infections could also be considered influencing factors, cervical human papillomavirus (HPV) infection is the main cause of cervical cancer. Metagenomics have been employed in the NGS era to study the microbial community in each habitat. Thus, in this investigation, virome capture sequencing was used to examine the virome composition in the HPV-infected cervix. Based on the amount of HPV present in each sample, the results revealed that the cervical virome of HPV-infected individuals could be split into two categories: HPV-dominated (HD; ≥60%) and non-HPV-dominated (NHD; <60%). Cervical samples contained traces of several human viral species, including the molluscum contagiosum virus (MCV), human herpesvirus 4 (HHV4), torque teno virus (TTV), and influenza A virus. When compared to the HD group, the NHD group had a higher abundance of several viruses. Human viral diversity appears to be influenced by HPV dominance. This is the first proof that the diversity of human viruses in the cervix is impacted by HPV abundance. However, more research is required to determine whether human viral variety and the emergence of cancer are related.

## 1. Introduction

Persistent infection with HPV, particularly high-risk HPV (hrHPV), is a critical point in the progression of cervical intraepithelial neoplasia (CIN), which leads to the development of cancer [1]. Cervical cancer is the fourth most common malignancy among women worldwide, accounting for 7% of all cancer cases [2,3]. The risk of acquiring any type of HPV infection during a woman’s lifetime is approximately 80%. Meanwhile, the risk of cervical cancer development is approximately 0.6% [4]. 

Besides HPV infection, the main factor of cervical cancer development, other factors also contribute to vaginal health and disease progression. Smoking has been listed as a co-factor of cervical cancer by International Agency for Research on Cancer (IARC) since 2004 [5,6]. The vaginal microenvironment plays a critical role in vaginal health by promoting the vagina homeostasis and inhibiting pathogenic bacterial infection [7]. It has been reported that the most important microorganism in the vagina is lactobacilli, which have the ability to promote a protective environment in the vagina via the production of metabolites, bacteriocins, and hydrogen peroxide (H_2_O_2_), and reduce the pH through the production of lactic acid [8,9,10,11,12]. However, the prevalence of microbial communities varies among different ethnic groups [13,14,15]. Many research teams investigated the link between the microbiome and cervical carcinogenesis in the vagina, but only a few studies have focused on the cervix, where HPV infection results in cervical cancer. According to research by Klein C. et al., the variety of bacteria in HPV-negative cervixes was less than that of HPV-positive cervixes [16]. Additionally, bacteria richness in the vagina and cervix was positively connected with the stage of cervical cancer development, although the amount of fungal variety was considerably lower in low-risk HPV (lrHPV)-infected samples than in hrHPV-infected samples [17].

The relationship between HPV and other viral infections in the cervix has been observed in many studies. Herpes simplex virus type 2 (HSV-2) seropositive patients had a two- to nine-fold increased risk of cervical squamous cell carcinoma or adenocarcinoma [18]. Approximately 27% of CIN patients had hrHPV and HSV-2 co-infections [19]. However, the lack of an association between HSV and cervical cancer has been revealed [20]. BK polyomavirus (BKV), a member of the family *polyomaviridae*, has been suggested as a contributor towards co-infection with HPV in the initiation and progression of cervical cancer, since BKV was found in 4% of HPV-positive cervical swabs [21]. In addition, HPV-Epstein Barr Virus (EBV) co-infection was also detected in cervical lesions [22]. With the technological advances thus far, NGS has been used to explore the viral community in the cervix. Siqueira JD. et al. revealed that the viruses in the families *Papillomaviridae, Anelloviridae, Genomoviridae,* and *Herpesviridae* were found (86%, 1%, 2%, and 0.06%, respectively) in the cervix of HIV/HPV co-infected patients in Brazil [23]. To the best of our knowledge, however, the virome in an HPV-infected cervix has not yet been studied. Hence, the aim of this study is to explore the viral communities in the cervixes of HPV16-infected patients.

## 2. Materials and Methods

### 2.1. Sample Selection

Cervical swab samples were collected from patients who visited the Department of Gynecological Outpatient, King Chulalongkorn Memorial Hospital, Bangkok, Thailand from 2019–2020 by gynecologists using colposcopy; the samples were stored at −80 °C. The Cobas 4800 high-risk HPV test (Roche, Switzerland) was used to detect the HPV genotyping in all samples. This assay was performed at the Virology Unit, Department of Microbiology, King Chulalongkorn Memorial Hospital, Bangkok. 

In this study, the archived samples were selected according to inclusion and exclusion criteria. All data were fully anonymized before assessment. This study was approved by the Institutional Review Board (IRB) of the faculty of Medicine, Chulalongkorn University, a WHO-certified ethics committee (COA No.159/2020, IRB No. 760/62, 27 January 2020). The protocol of study was also approved by the Institutional Biosafety Committee (IBC) of the faculty of Medicine, Chulalongkorn University (MDCU-IBC018/2019). The blood-free samples with HPV infection (HPV16 and/or other hrHPVs) and histopathology results diagnosed as CIN1 to CIN3 were selected. The exclusion criteria included: having menstruation on the date of specimen collection, infecting with other active microorganisms (HIV infection, bacterial vaginosis, vaginal candidiasis, etc.), use of antibiotics or vaginal antimicrobials within the last month, and pregnancy. A total of 48 leftover HPV DNA positive specimens were recruited in this study. Due to the very low yield of nucleic acid, only 35 samples were suitable for further experiments. The MEAN ± SEM age of all 35 enrolled patients was 37.971 ± 1.459 years (ranging from 23 to 50 years old). The additional information of each sample, e.g., HPV genotype, cytological and histological characteristics, are shown in Table S1.

### 2.2. DNA/RNA Extraction from Cervical Specimens

The leftover cervical specimens were beaten by Pathogen Lysis Tubes L (Qiagen, Germany) at 30 Hz for 5 min using Tissue lyser LT (Qiagen, Germany). After that, the COBAS 4800 system (Roche, Switzerland) was used for the automatically total nucleic acid extraction. To recover the RNA viruses in samples, cDNA construction and the second strand synthesis step were performed; the cDNA was constructed by Roche Evoscript Universal cDNA Master using random hexamers (Roche, Switzerland) by heating at 65 °C for 30 min. After that, the second strand of cDNA was synthesized by using 10 U/µL DNA polymerase I, second Strand buffer (10×NEB Buffer 2), 10 mM dNTP mix, RNaseH, and 0.1 M DTT (New England Biolabs, UK) by incubating at 16 °C for 2.5 h. The cDNA/second strand was purified by AMPure XP beads and suspended in 33 µL of 10 mM Tris HCl pH 8.0 (Biobasic, Markham, ON, Canada).

### 2.3. Virome Capture Sequencing (VirCapSeq)

In the library preparation step, the guideline from SeqCap EZ Hypercap Workflow Version 2.3 (Roche, Switzerland), with slight modification, was used. All reagents, i.e., the KAPA HyperPlus Library Preparation Kit and SeqCap Adaptors Kit A, were purchased from Roche (Switzerland). In brief, the 100 ng of DNA input was enzymatically sheared by incubating at 37 °C for 15 min to obtain an average fragment size of 200 bp and followed by end repair, A-tailing reaction, and adaptor ligation. After that, double-sided size selection using AMPure XP beads and Pre-Capture LM-PCR was performed. The first QC step, including the measurement of yield and fragment size, was performed by using Nano Drop (the yield libraries should be ≥ 1.0 µg) and Fragment Analyzer 3700 (150 to 500 bp with a peak at 320 bp). To capture and enrich the viral sequences, 1 μg of each DNA sample library was hybridized with the SeqCap EZ probe pool (VirCapSeq-VERT Capture Panel); there were approximately 2.1 M probes (variable lengths of 50–105 mers) covering the genomes of 207 viral taxa [24], in a thermocycler at 95 °C for 5 min and 47 °C for 16 h. Then, the sample was mixed with pre-wash Capture Beads and incubated at 47 °C for 15 min. After that, the post-capture LM-PCR amplification was performed by using the following conditions: 98 °C for 45 s with 14 cycles of 98 °C for 15 s, 60 °C for 30 s and 72 °C for 30 s, then 72 °C for 1 min. Finally, the libraries were cleaned using AMPure XP beads. The final QC step included the measurement of yield and fragment size, which was performed using Nano Drop (the yield libraries should be around 500 ng) and Fragment Analyzer 3700 (150 to 500 bp).

### 2.4. Illumina Sequencing

Before sequencing, the yield of libraries was quantitated by the KAPA library quantification kit (Roche, Switzerland). The denature and dilute libraries guide for MiSeq System (Document # 15039740 v10; Illumina, San Diego, CA, USA) was used as a guideline for sample preparation. The 4 nM of normalized libraries was adjusted to 18 pM and loaded onto the reagent cartridge for sequencing using a MiSq Reagent Kit v3 (Illumina, San Diego, CA, USA). The libraries were sequenced using the Illumina MiSeq platform to generate 2 × 300 bp reads.

### 2.5. Data Analysis

The raw data in FASTQ files were exported from the Illumina MiSeq. To identify the metadata of viral species, these raw data were analyzed by the Virus Identification Pipeline (VIP) through sense mode, which function on Docker and Ubuntu 20.04 LTS. This pipeline was developed by Li Y. et al. [25]. For the QC step, the DUST algorithm was used for trimming the adapter and low-quality reads, while the low-complexity sequences were removed. The trimmed reads lengths more than 20 bp were retained by using PRINSEQ [26]. The Bowtie2 was used to subtract the host-related reads. The bacteria and related ribosomal RNA (rRNA) reads were removed, and the remaining reads were aligned to the virus database. After that, unmatched reads were re-aligned to a viral protein database from NCBI Refseq DB using RAPSearch. Finally, all matched reads were classified under a genus for de novo assembly and phylogenetic analysis. All virus databases were downloaded from ftp://ftp.ncbi.nih.gov/refseq/release/viral/ (accessed on 11 July 2020). All scripts for VIP were provided in Supplementary Method S1.

### 2.6. Statistics

Alpha (Shannon index) and beta diversity, as well as principal coordinate analysis (PCoA) based on Bray-Curtis dissimilarities of the microbial community structure, were calculated by Past 4.03 software; meanwhile, the boxplots, rarefaction curve, bar chart, and pie chart were constructed by GraphPad Prism 8 software (Dotmatics, San Diego, CA, USA). Mann-Whitney testing (non-parametric Unpaired t test) was used to determine the significant difference of the alpha diversity and relative abundance.

## 3. Results

### 3.1. Diversity of Viruses in the Cervix

A total of 35 cervical samples from colposcopy were recruited. They were separated into two groups based on the results of the histological analysis: CIN1 (*n* = 21) and CIN2/3 (*n* = 14). Of those samples, 14 samples had HPV16 infection exclusively, 14 samples had HPV16 infection combined with other hrHPVs, and 7 samples had other hrHPV infection (Table S1).

**Figure 1:**
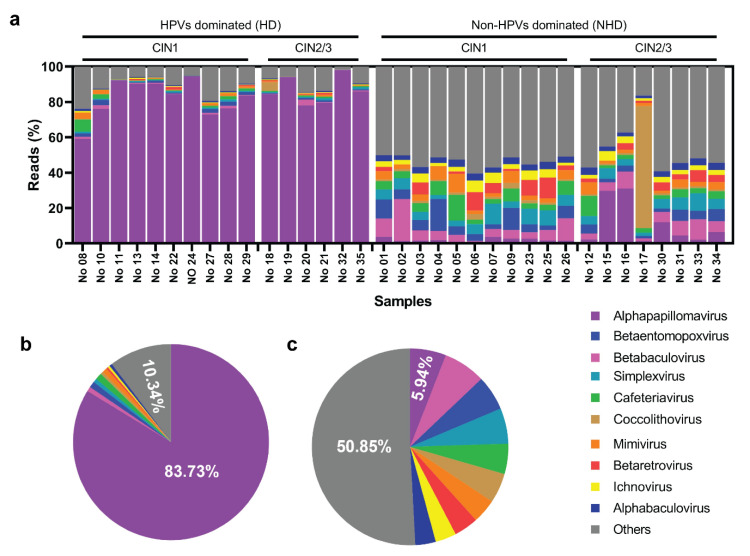
Global taxonomic pattern of viral abundance for the dominant genera in the cervix. (**a**) The percentage of viral reads in each cervical sample, HPV-dominated (HD; left) and non-HPV-dominated (NHD; right). (**b**,**c**) Pie graph representing the means of viral reads (%) in HD and NHD groups.

The 1.5 M (1,514,093) of viral reads with 43,260 mean of viral reads per sample were subtracted from 8.5 M (8,562,489) high-quality reads by VIP [25]. A total of 602 species and 350 genera of viruses were found in the cervical samples. Figure 1a displayed the top 10 virus genera (ordered by abundance). *Alphapapillomavirus*, of which HPV is a member, was the most dominant genus in the cervix, followed by *Betatomopoxvirus, Betabaculovirus, Simplexvirus, Cafeteriavirus, Coccolithovirus, Mimivirus, Betaretrovirus, Ichnovirus,* and *Alphabaculovirus*. HPV16, 32, and 53 were the most dominant among all samples. Using the percentage of HPVs abundance as a criterion, the samples were divided into two groups: HPV-dominated (HD) and non-HPV-dominated (NHD) (Figure 1a). In the HD group (*n* = 16), the average relative abundance of HPVs was 83.73% (ranging from 60% to 98%) (Figure 1b). Only 5.94% (ranging from 1% to 31%) of HPV relative abundance was found in NHD groups (*n* = 19) (Figure 1c); meanwhile, no significant association of ages between HD (35.188 ± 2.326 years) and NHD (40.316 ± 1.715 years) groups was found (*p* = 0.1217; Mann-Whitney U-Test). The genome coverage of *Alphapapillomavirus* ranged from 83.68% to 100% in the HD group, and from 24.27% to 99.75% in the NHD group. Samples in each histological stage of HD and NHD groups were CIN1 47.62% and 52.38%, and CIN2/3 42.86% and 57.14%, respectively. Interestingly, besides humans, viruses from other hosts, e.g., vertebrate viruses, were also found. However, due to the specificity of the viral infection, we only focused on the human-related viruses.

**Figure 2:**
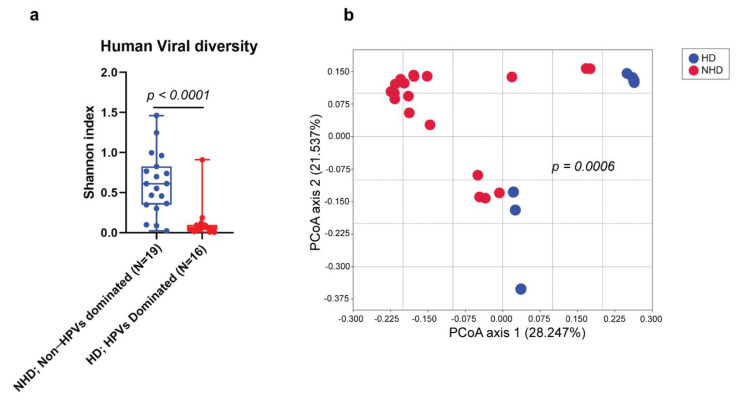
Viral diversity in HD and NHD groups. (**a**) Shannon (Alpha) diversity, (**b**) β-diversity using principal coordinate analysis (PCoA) based on Bray-Curtis dissimilarities. Error bars represent the standard error of mean (SEM).

In this study, alpha diversity for the observation of human viral diversity within an individual sample was performed by using the Shannon index (estimated evenness and richness). The alpha diversity of viruses in the NHD group was statistically higher than that of the HD group (Figure 2a). The same trend of statistical difference was found in beta diversity when using PCoA analysis (Figure 2b). These results indicated that the viral communities in NHD cervixes contained greater diversity than those in HD cervixes. 

### 3.2. The Virome Composition in Cervical Specimens

**Figure 3:**
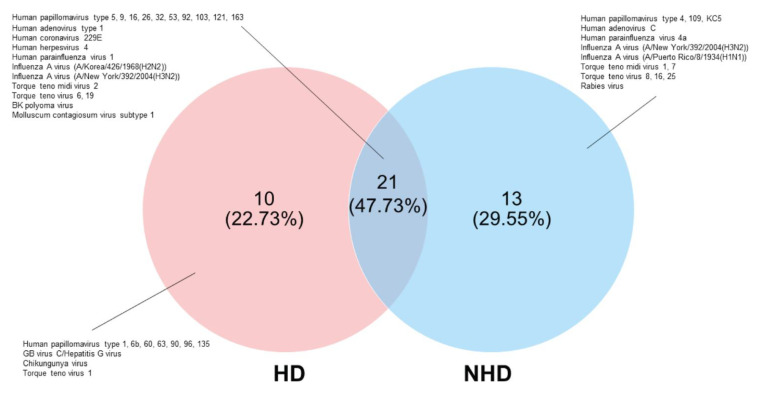
The unique and shared viral genera in the cervical samples. Venn diagrams showed numbers of unique and shared OTU (species) of human viruses between the HD and NHD groups.

As demonstrated in Figure 3, a Venn diagram represents the unique and shared human virome in the cervixes of HD and NHD groups. A total of 21(47.73%) viral species were shared between HD and NHD groups. The human viruses which were found in this intersected group were HPVs (type 16, 9, 32, 92, 121, 103, 163, 5, 53, 26), influenza A virus (H3N2), human coronavirus 229E (HCoV-229E), torque teno midi virus (TTMDV) type 2, human parainfluenzavirus 1 (HPIV-1), TTV (type 19 and 6), BKV, human adenovirus type 1 (HAdV-1), MCV and HHV4 (EBV). The unique human viruses in the HD group (22.73%) included HPVs (type 1, 135, 63, 60, 96, 6b, and 90), hepatitis G virus, TTV1, and chikungunya virus, whereas HPVs (type 4, KC5, and 109), TTV (type 8, 16, and 25), human adenovirus C, human parainfluenza virus 4a (HPIV-4a), influenza A virus (H1N1), rabies virus, and TTMDV (type 1 and 7) were unique in the NHD group (29.55%). 

**Figure 4:**
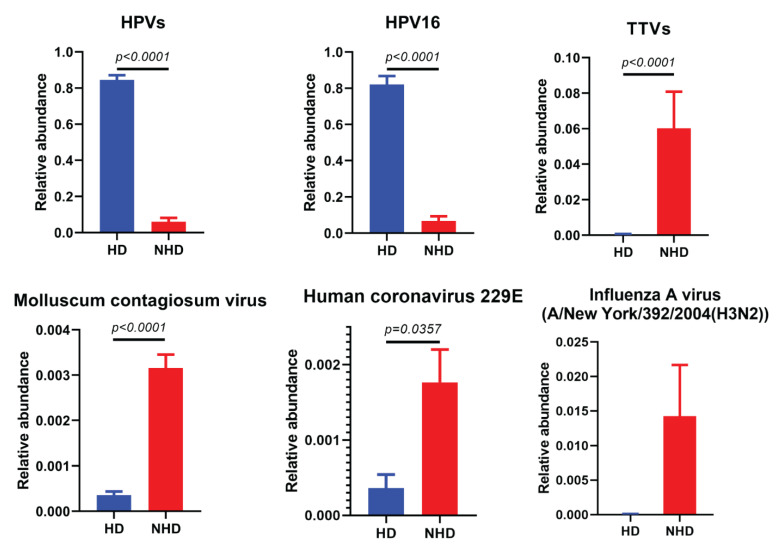
Relative abundances of selected taxa of human viruses between HD and NHD groups. Error bars indicate the standard error of mean (SEM). Mann-Whitney U testing for non-parametric data was used for comparison between the abundance of each group.

The relative abundance of human viruses between groups was calculated. As predicted, the relative abundance of HPVs and HPV16 in HD group was significantly higher than that of the NHD group. On the other hand, the relative abundances of TTVs, HCoV-229E, and MCV were found to be significantly higher in the NHD group when compared to the HD group (Figure 4), whereas the relative abundances of the other human viruses were not different.

### 3.3. Abundance and Ubiquity of Virome in Cervix among HD and NHD Groups

**Figure 5:**
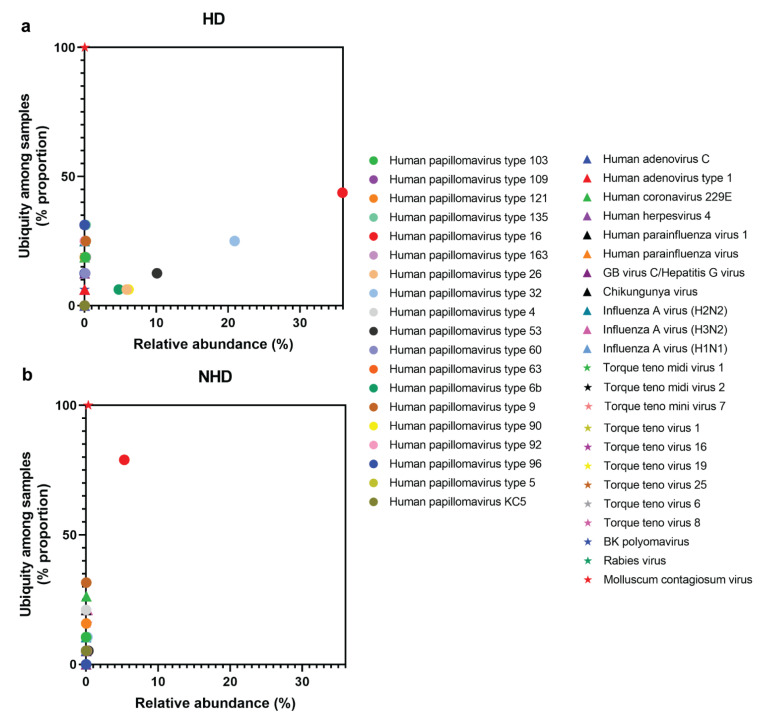
Ubiquity dot plots representing viral species in the cervix between HD (**a**) and NHD (**b**) groups. The graphs were plotted between the mean of ubiquity (y axis) and relative abundance (x axis) from each species. (● =HPVs, ▲ and ★ = Other human viruses).

The most dominant viral species in the HD group was HPV16 (~30% abundance and ~50% proportion), followed by HPV 32 and 53 (Figure 5). The relative abundance of other human viruses was low (less than 10%) when compared to HPVs, but the proportion of those viruses was varied. For example, the proportions of influenza A virus (H2N2), HCoV-229E, and influenza A virus (H3N2), were 25.00%, 18.75%, and 12.50%, respectively. In the NHD group, HPV16 was also the most dominated viral species (only ~5% abundance and ~80% proportion), followed by HPV 9 and 4. The proportions of other human viruses, i.e., HCoV-229E, influenza A virus (H3N2), and TTV8, were 26.32%, 21.05%, and 15.79%, respectively. Interestingly, MCV was detected in all cervical specimens with a 100% proportion in both groups.

## 4. Discussion

The virome in an HPV-infected cervix was investigated. A diverse viral community was discovered (Figure 1). As predicted, HPVs were the most abundant in most cervical samples. Besides HPVs, other human viruses were discovered, such as influenza A virus (H1N1, H2N2, H3N2), HCoV-229E, TTVs, TTMDVs, HPIVs, BKV, HAdV-1, MCV, and HHV4. Figure 1 demonstrated that the samples were divided into two groups based on the domination of HPVs, and the viral diversity (alpha and beta) was controlled by the domination of HPVs (Figure 2). These results revealed that with greater occurrences of HPVs, the less frequently other viruses were found. 

During viral infections, many cellular mechanisms such as molecular building blocks, e.g., nucleotides and amino acids, a source of chemical energy, and the cellular protein-synthesizing machinery, are hijacked by viruses to support their own replication cycle and express their specific proteins [27]. Once one virus infects the cells, the interference phenomenon might occur. Hence, the following viruses may not be able to infect the cells, or infection may occur but without replication. In addition, the competitive infection during the co-infection model of viruses, which have different life cycle times, was demonstrated in Vafadar S. et al. [28]. Moreover, immunity to viral infection also possesses a critical role in protecting against viral infection. Viral-infected cells secrete type I interferon (Type I IFN) to induce the expression of enzymes that block viral replication, leading to the initiation of an antiviral state in uninfected cells [29]. As a result, we hypothesized that the more HPV is present, the more the antiviral state is activated, resulting in a lower variety of other viral infections. Several immune evasion mechanisms were found in HPV, but not in many other viruses [30,31,32,33,34,35,36]. This is possibly due to the persistent property of HPV. Altogether, these results might explain the possibility of dominated HPV infection affecting other viruses in the same environment, such as the cervix.

Besides HPVs, many species of human viruses were shared between HD and NHD groups, e.g., HHV-4, BKV, TTV, and MCV (Figure 3). Although the prevalence of 25% of HPV and HSV coinfection was previously reported in cervical samples [19], no read of HSV was detected in this study. Nevertheless, HHV-4 or EBV was detected. EBV is a ds-DNA virus which causes infectious mononucleosis, multiple lymphoid and epithelial malignancies, e.g., B-cell lymphomas (Burkitt lymphoma), and various T-cell/NK lymphoproliferative disorders [37]. This virus typically infects epithelial cells and B cells, establishing lifelong persistent infection, while the latency stage of this virus is at memory B cells. However, it has been reported that EBV can infect the cervical epithelial cells [38]. Interestingly, in this study, EBV was found in both the HD and NHD groups at the same abundance and ubiquity (Figure 5). A possible function for EBV in the development of cervical cancer was postulated in a recent publication that discovered EBV in HPV-infected cervical tissues. According to Landers RJ et al. and Sasagawa T et al., the EBV DNA were detected in both precancerous (CIN) and invasive cervical cancer cells [39,40]. The risk of cervical cancer was found to be enhanced four-fold in HPV-EBV-positive women [41]. EBV oncoproteins EBNA-2 and LMP1 were found in high-grade CIN lesions and cervical cancer cells [40]. In the female reproductive period, EBV infection may facilitate chronic cervicitis [42]. Once EBV arrives at the cervix, this virus may accelerate the integration of the HPV genome into the host cell, leading to genomic instability in the HPV-infected cervical cells [37]. Co-infection of EBV and HPV may influence each other’s life cycles. Degradation of retinoblastoma protein by HPV E7 activates cell cycle progression independent of p16 inhibition of cyclin D/cyclin-dependent kinase complexes, leading to the establishment of EBV latency [43]. Moreover, the dramatic reduction of EBV DNA replication was found in HPV culture using the human organotypic rafts system, suggesting that the EBV latency was promoted by HPV [43].

BKV belongs to the family *Polyomaviridae*. Humans are the hosts of this virus [44]. This virus shares structural and functional similarities with papillomaviruses. The circular ds-DNA genome was packed into the naked icosahedral capsid. BKV normally infects epithelial cells in various parts of the body. It can either infect permanently as a latent state or shed via urine and saliva. It has been reported that polyomaviruses contain the oncogenic function, i.e., the large T antigen, which plays a role in carcinogenesis in the same way as HPV E6 and E7 [43]. The correlation of HPV/BKV co-infection and cervical cancer has been potentially revealed. BKV DNA was discovered in 35% of hrHPV-infected high-grade squamous intraepithelial lesions (HSIL), while there was no BKV DNA detected in low-grade lesion (LSIL) or normal tissue, suggesting that BKV could contribute to cancer development [45]. 

The family *Anelloviridae* is classified as a circular negative ss-DNA virus. Their genome is contained in an icosahedral capsid without an envelope [46]. This family is divided into many species, e.g., TTV, Torque teno mini virus (TTMV), and TTMDV. Almost 70% of blood viromes contain anelloviruses (AVs) and more than 90% of these AVs are TTV, which is in the genus *Alphatorquevirus* [47]. TTV is transmitted through multiple routes, including respiratory, placental, and transfusion, at a very early stage of life [48]. It is mainly replicated in T lymphocytes in vivo [49]. Hence, the prevalence of TTV was found to be up to 95% in humans worldwide, which was independent of age, health conditions, and socioeconomic standing [50]. To this date, human TTVs have been used as a biomarker for predicting anthropic pollution and immunity statuses [50]. Some evidence revealed that TTV viremia correlated well with the intensity of maintenance immunosuppression in orthotopic liver transplant recipients [51]. Interestingly, anellovirus was also found in 12% of HIV/HPV co-infected cervixes [23]. Nevertheless, the role of TTV in any clinical manifestation is still unknown.

MCV is an enveloped, linear, ds-DNA genome which is categorized in the family *Poxviridae.* MCV infection produces benign raised bumps, or lesions, on the upper layers of the skin. The co-infection of MCV and HPV was previously suggested. However, the investigation of this coexistence failed [52]. Hence, this is the first study to demonstrate MCV/HPV co-infections in cervical samples utilizing the NGS technique. 

In this study, many human respiratory viruses were found in the cervix, such as influenza A virus, HAdV-1, HCoV-229E, and HPIVs. We hypothesized that these viruses were transmitted to the cervix via oral sexual behavior. In addition, it is possible that some viruses might not infect the cells but only the surface or contact of the genetic material residues. Further study is required, since the role of these respiratory viruses in carcinogenesis and their relationships with HPV co-infection is still unknown.

## 5. Conclusions

The present study is the first study to indicate that the domination of HPVs possesses an important role in human viral diversity. Since HPV infection plays an important role in cancer development, therefore, co-infection with other viruses might act as cofactors in HPV related carcinogenesis. Nevertheless, the route of transmission and the role of pathogenicity of these viruses are still unclear. It would be valuable to conduct follow-up studies to ascertain the involvement of HPV co-infection with various viral species in the development of cervical cancer. An experiment using human organotypic raft culture could be an effective way to demonstrate the role of those viruses in HPV co-infection. The increasing number of clinical specimens should be further investigated to confirm the virome composition. In addition, more thorough investigations with various control groups, such as normal and/or CIN1-3 HPV negative groups, should be planned in future studies to confirm the function of HPVs in the virome composition. As a result, regardless of population, HPV persistence is a dominant factor in cervical cancer progression, though the microbial community may differ in other demographic populations. Therefore, the virome study in other ethnic groups should be further conducted.

## Data Availability

Raw sequence files from virome capture sequencing were deposited into the NCBI Sequence Read Archive (SRA) under accession numbers PRJNA766412 (Available after publishing).

## Supplementary Materials

The following supporting information can be downloaded at: https://www.mdpi.com/article/10.3390/v14092066/s1, Table S1: The general information of each cervical sample, Supplementary Method S1: The scripts for Virus Identification Pipeline.
