# Supplementary material for: Human Virome in Cervix Controlled by the Domination of Human Papillomavirus
Source: Viruses. 2022 Sep 17;14(9):2066. doi: 10.3390/v14092066 (PMC9503738; doi:10.3390/v14092066)
Supplement: Supplementary file 1 [file viruses-14-02066-s001.zip › viruses-1918588-supplementary.pdf]

Table S1. The general information of each cervical sample.

Patient no.	Histology	HPV Genotype	Cytology	Age
**1**	CIN1	HPV16	ASC-US	48
**2**	CIN1	HPV16	LSIL	41
**3**	CIN1	HPV16	HSIL	38
**4**	CIN1	HPV16	LSIL	40
**5**	CIN1	HPV16	LSIL	48
**6**	CIN1	HPV16	HSIL	41
**7**	CIN1	HPV16	ASC-H	48
**8**	CIN1	HPV16	LSIL	50
**9**	CIN1	HPV16	LSIL	26
**10**	CIN1	HPV16 and other hrHPVs	LSIL	36
**11**	CIN1	HPV16 and other hrHPVs	LSIL	23
**12**	CIN2	HPV16 and other hrHPVs	HSIL	46
**13**	CIN1	HPV16 and other hrHPVs	LSIL	45
**14**	CIN1	HPV16 and other hrHPVs	LSIL	44
**15**	CIN3	HPV16	HSIL	27
**16**	CIN2	HPV16	ASC-US	34
**17**	CIN3	HPV16	ASC-H	35
**18**	CIN3	HPV16	LSIL	34
**19**	CIN2	HPV16 and other hrHPVs	LSIL	36
**20**	CIN2	HPV16 and other hrHPVs	ASC-US	23
**21**	CIN3	HPV16 and other hrHPVs	ASC-US	32
**22**	CIN1	HPV16 and other hrHPVs	NILM/LSIL	28
**23**	CIN1	HPV16 and other hrHPVs	LSIL	49
**24**	CIN1	HPV16 and other hrHPVs	ASC-US	36
**25**	CIN1	HPV16 and other hrHPVs	LSIL	32
**26**	CIN1	Other hrHPVs	LSIL	39
**27**	CIN1	Other hrHPVs	LSIL	44
**28**	CIN1	Other hrHPVs	ASC-US	23
**29**	CIN1	Other hrHPVs	ASC-US	36
**30**	CIN3	HPV16	HSIL	49
**31**	CIN2	HPV16 and other hrHPVs	ASC-H	42
**32**	CIN3	Other hrHPVs	LSIL	24
**33**	CIN3	Other hrHPVs	ASC-H	49
**34**	CIN2	Other hrHPVs	LSIL	34
**35**	CIN3	HPV16 and other hrHPVs	LSIL	49

Supplementary Method S1. The scripts for Virus Identification Pipeline

1. Copy local files into docker
   docker cp /mnt/[PATH]/[FILE NAME_R1&2].fastq.gz [CONTAINER ID]:/
2. Go to the container
   sudo docker exec -it [CONTAINER ID] bash
3. Extract the FASTQ files

*$ gunzip *.gz*

4. Merge the read1 and read2 files
   $ cat [FILE NAME_R1.fastq] [FILE NAME_R2.fastq] >[FILE NAME.fastq]
5. Run the VIP (Using sense mode)
   $ VIP.sh -z -i [FILE NAME.fastq] -p illumina -f fastq -r /VIPDB
   $ /VIP/VIP.sh -c [FILE NAME.fastq].conf -i [FILE NAME.fastq]
6. Export the outputs
   docker cp [CONTAINER ID]:/[FILE NAME] /mnt/[PATH]
